# Ovarian somatic tissue rejuvenates circulating apolipoproteins and promotes cognitive health in postreproductive female mice

**DOI:** 10.1007/s11357-025-02019-4

**Published:** 2025-12-13

**Authors:** Nathan D. McCoy, Steven P. Gawrys, Samuel G. Mackintosh, Stephanie Byrum, Negar Kosari, Anhong Zhou, Mishfak A. M. Mansoor, Yuji Ikeno, José V. V. Isola, Michael B. Stout, Augusto Schneider, Michal M. Masternak, Jeffrey B. Mason

**Affiliations:** 1 College of Veterinary Medicine, Department of Veterinary Clinical and Life Sciences, Center for Integrated BioSystems, Utah State University, 4700 Old Main Hill, Logan, UT 84322 USA; 2 University of Florida, Community Health and Family Medicine, Gainesville, FL 32608 USA; 3 IDeA National Resource for Quantitative Proteomics, Little Rock, AR 72205 USA; 4 Barshop Institute for Longevity and Aging Studies, The University of Texas Health Science Center at San Antonio, San Antonio, TX 78229 USA; 5 Department of Pathology, The University of Texas Health Science Center at San Antonio, San Antonio, TX 78229 USA; 6 Geriatric Research and Education Clinical Center, Audie L. Murphy VA Hospital, South Texas Veterans Health Care System, San Antonio, TX 78229 USA; 7 Aging and Metabolism Research Program, Oklahoma Medical Research Foundation, Oklahoma City, OK 73104 USA; 8 Faculdade de Nutrição, Universidade Federal de Pelotas, Pelotas, RS Brazil; 9 College of Medicine, Burnett School of Biomedical Sciences, University of Central Florida, Orlando, FL USA; 10 Department of Head and Neck Surgery, Poznan University of Medical Sciences, Poznan, Poland

**Keywords:** Apolipoproteins, Ovarian aging, Menopause, Health span, Alzheimer’s

## Abstract

Women experience more pronounced lipidomic changes with aging than men, which may contribute to the higher rates of Alzheimer’s disease seen in postmenopausal women. Our earlier findings showed that transplantation of young ovarian somatic tissues or cells produced positive health-enhancing results in postreproductive females. In the current experiments, we looked to find key health-enhancing ovarian cells and pathways involved in this phenomenon. We conducted physiological and molecular analysis on animals/samples from old, postreproductive mice that received young ovarian tissue/cell transplants. Our analysis revealed a loss with age and a restoration with ovarian tissue/cell exposure, of serum biomarkers of lipid signaling and histological and behavioral markers of cognitive function. We further found, with single-cell transcriptomics and Raman spectroscopy, two candidate ovarian somatic cell types implicated in the restoration of health through a lipid signaling-based process. These results have identified key factors toward the determination of how germ cell-independent ovarian somatic tissues restore health through regulation of lipid signaling and dementia in postreproductive female mice.

**Graphical Abstract:**

**Figure:**
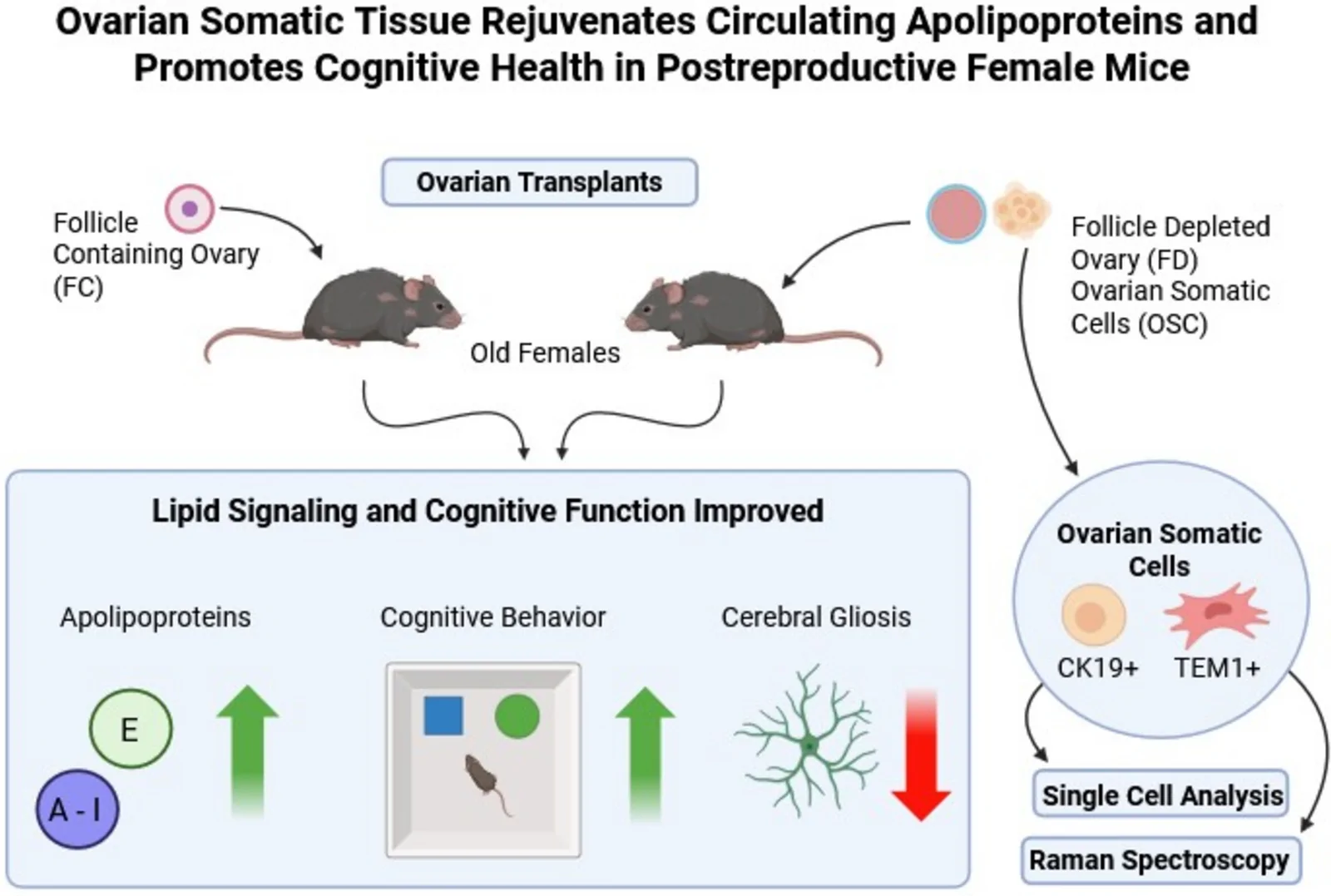

## Introduction

Women experience more pronounced lipidomic changes with aging than men, which may contribute to the higher rates of Alzheimer’s disease observed in postmenopausal women [1, 2]. In women, lipoprotein factors appear to change in response specifically to reproductive aging rather than chronological aging [3]. Apolipoproteins play a critical role in lipid metabolism [4–7] and in the development and progression of Alzheimer’s disease in both animal models [8] and in humans [9, 10].

While laboratory studies often highlight the cognitive benefits of hormone therapy, these effects have not consistently been replicated in human studies [11, 12]. Some studies found that hormone therapy can increase the risk of dementia and cognitive decline [13], is associated with elevated Alzheimer’s-related neurofibrillary tau tangles [14], and may promote breast cancer and melanoma and reduce immune health [15]. However, it remains unclear how menopausal alterations in lipid transport, metabolism, and signaling impact disease progression in women [16, 17] and if these changes are solely dependent on ovarian hormonal output.

Our previous findings suggest that health in postreproductive females can be restored by germ cell-independent ovarian somatic tissues [18–24]. Transplantation of young ovarian somatic tissues or cells produced positive health-enhancing results in postreproductive females. Isolated ovarian somatic cells can still produce hormones but do so differently when separated from germ-cell influence and in an aged, postreproductive environment/niche. Identifying the functional roles of these candidate somatic cell populations and connecting the molecular/signaling pathways should help determine the identities of key health-enhancing ovarian factors. In the current experiments, we leveraged previous ovarian single-cell experiments, which provided critical insights into mechanisms responsible for ovarian aging phenotypes and successfully identified several age-associated transcriptomic changes in the aging mouse ovary, including age-related changes in stromal cell regulation [25]. The ovarian surface epithelium is believed to be the origin of 85–90% of high-grade serous ovarian cancers. The ovarian stroma is the gatekeeper of blood, nutrients, and signaling entities entering and exiting the ovary, can hypertrophy in PCOS, and is the source of sex cord-stromal tumors [26, 27]. An interaction between the ovarian surface epithelium (CK19+) and the ovarian stroma (TEM-1+) may also contribute to ovarian cancers [28].

Female reproductive health is associated with a longer lifespan, but familial longevity is not associated with extended reproductive health. Furthermore, there is no significant association between familial longevity and genetic predisposition to age at menopause, suggesting that additional, non-genetic ovarian somatic factors contribute to a longer lifespan [29]. We proposed that somatic CK19+ ovarian surface epithelium cells and TEM-1+ stromal cells would produce divergent signatures and contribute differently to the health-restoration effects demonstrated with transplantation of the entire young ovarian somatic cell population. Given that hormones account for only 10% of menopausal lipid changes [30], in the current study, we investigated how germ cell-independent ovarian somatic tissues further regulate lipid signaling and dementia in postreproductive female mice.

## Materials and methods

### Animals

The present study used CBA/J and C57BL/6 mouse strains as aging models for postreproductive health [19, 31, 32]. Female mice were obtained from the National Institute of Health-National Institute of Aging (Bethesda, MD, USA) and Jackson Laboratory (Bar Harbor, ME, USA). Each mouse was individually housed in ventilated cages (Green Line IVC Sealsafe Plus, Techniplast, West Chester, PA, USA) containing corncob bedding (7097 Corncob, Harlan Teklad, Bartonville, IL, USA). Each home cage had deionized water, laboratory rodent diet ad libitum (2018 Teklad Global 18% Protein Rodent Diet, Harlan Teklad, Bartonville, IL, USA), and added enrichment (4 cm dia × 10 cm L paper tubes, nestlets, kimwipes). All females were exposed to male mice (no more than 2 cages away from a cage containing a male/males)/male bedding (a small amount of soiled bedding from a male’s cage was mixed with fresh bedding at cage changes) throughout the study. The colony environment had fresh filtered air (15 changes/hour), regulated temperatures of 21 ± 2 °C, humidity of 50 ± 20%, and an even light-dark cycle (12:12 h) and was located in the Utah Science, Technology and Research Center (USTAR) at Utah State University. USTAR is an American Association for Accreditation of Laboratory Animal Care (AAALAC) approved facility in accordance with the National Institutes of Health and Animal Use guidelines. Animal care protocols were developed under the National Research Council guidelines found in the Guide for the Care and Use of Laboratory Animals. Protocols were approved by the Utah State University Institutional Animal Care and Use Committee (IACUC-10222). To provide context for gliosis severity, additional samples from a previous experiment with untreated 7-month-old APPswe/PSEN1dE9 (APP/PS1) double-transgenic Alzheimer’s disease mice with a B6 × C3 background (MMRRC stock No. 034829-JAX) were included in the processing of brain samples for assessment of gliosis.

### Experimental design

**Fig. 1:**
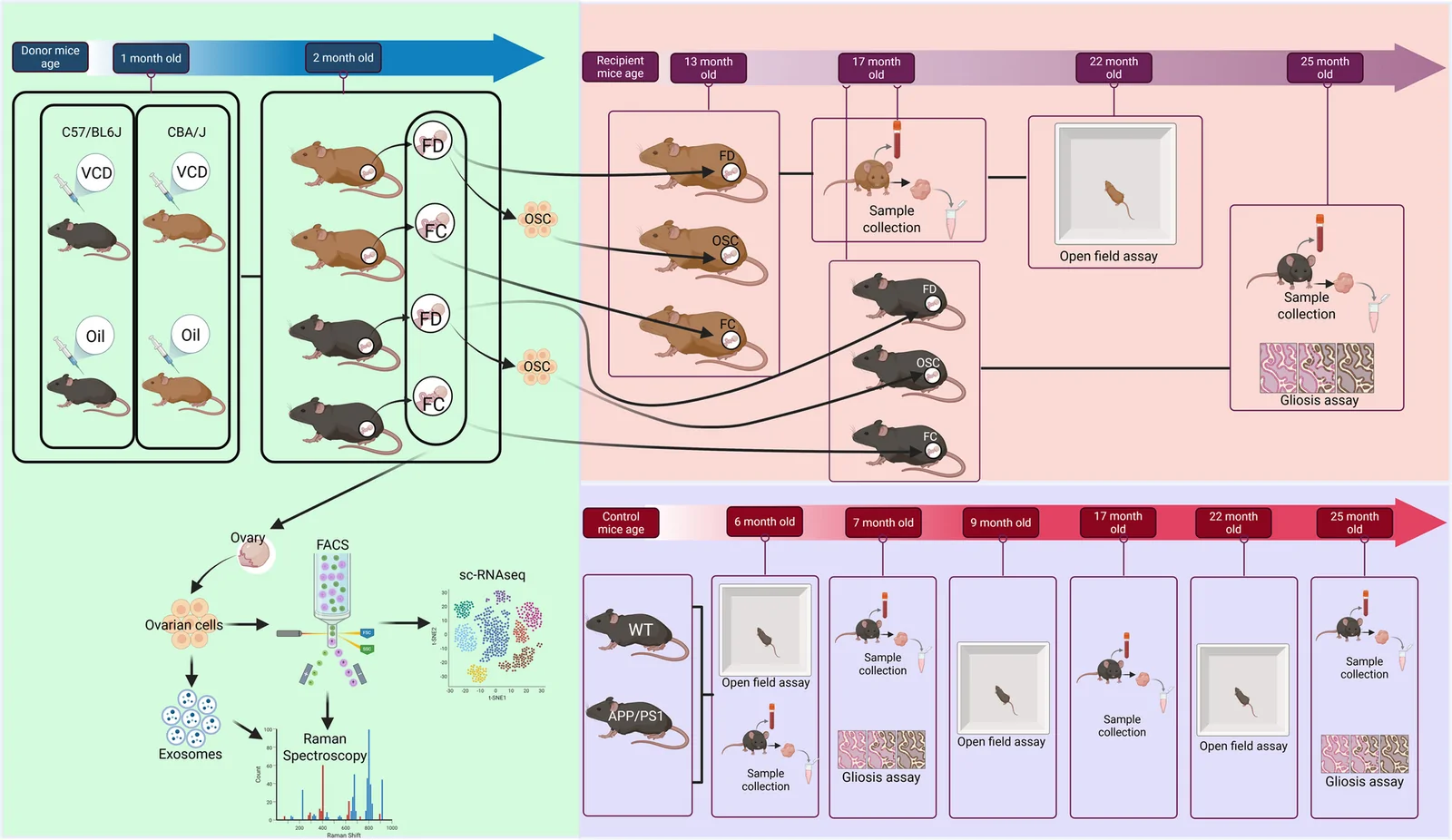
Experimental design. Donor CBA/J and C57BL/6 mice received treatment with 4-vinylcyclohexene diepoxide (VCD) or vehicle (OIL) for 28 days. Ovarian tissues/cells from 60-day donor mice were collected, processed, and transplanted to 13-month CBA/J and 17-month C57BL/6 recipient mice. Cells from 60-day CBA/J mice were used for single-cell and Raman analysis. CBA/J mice were analyzed for proteins at 6 and 17 months of age and health span assays at 22mo. C57BL/6 mice were assayed for gliosis at 7mo and 25mo. Samples from untreated APP/PS1 mice from a different experiment were included to provide histological and quantitative context in the gliosis assays. OIL = animal treated with sesame oil (vehicle) only; FD = follicle-depleted ovary; FC = follicle-containing ovary; OSC = isolated ovarian somatic cells; CTL = control. BioRender

Animals were randomly assigned to control or experimental groups (Fig. 1). Control groups consisted of mice with their original ovaries intact (not sham-operated). In previous experiments, sham-operated mice were no different from unoperated mice in terms of lifespan, cardiomyopathy, or osteoarthritis [32–34]. Experimental groups consisted of CBA/J and C57BL/6 females who received ovarian tissue/cell transplants. CBA/J mice at 13 months of age who were assessed as reproductively senescent (acyclic) via vaginal cytology, received, from 60-day CBA/J donor mice, a pair of germ cell/follicle-containing (FC) 60-day ovaries, germ cell/follicle-depleted (FD) 60-day ovaries, or a direct intraovarian injection (10 ul; ~ 11k cells) of an ovarian somatic cell (OSC) single-cell suspension directly into both endogenous ovaries. C57BL/6 mice at 17 months of age who were assessed as reproductively senescent (acyclic) via vaginal cytology, received, from 60-day C57BL/6 donor mice, a pair of germ cell/follicle-containing (FC) 60-day ovaries, germ cell/follicle-depleted (FD) 60-day ovaries, or a direct intraovarian injection (10 ul; ~ 11k cells) of an OSC single-cell suspension directly into both endogenous ovaries. Samples from untreated 7-month APP/PS1 mice were obtained from a previous study (Dr. Ikeno-unpublished) to provide context for the quantitative and histological gliosis analysis.

### Surgical procedures

Surgical procedures included anesthetics for both donor and recipient mice and postoperative administration of analgesia with extended administration of analgesia if necessary. Euthanasia of donors and recipient mice occurred by cervical dislocation followed by thoracotomy with rapid exsanguination by cardiocentesis. Mice were monitored twice daily and more frequently if health concerns arose [32].

### Ovarian tissue/cell transplantation

CBA/J mice at 13 months of age and C57BL/6 mice at 17 months of age underwent a bilateral ovariectomy and bilateral ovarian transplantation with a pair of ovaries or ovarian cells from 60-day donor mice of the same strain. Bilateral ovarian transplantations were performed as previously described [32, 33, 35]. Briefly, recipient and donor mice were anesthetized using an intraperitoneal injection of an anesthetic cocktail (100 mg/kg ketamine, 20 mg/kg xylazine, and 10 mg/kg acepromazine). The ovaries of the donor mice were exposed starting with a paralumbar incision. Once through the skin and peritoneum, the ovarian fat pad, located distal to the kidney, was placed into the field of view and positioned with a clamp to expose the ovarian bursa containing the ovary. The ovary was then removed by incising the bursa opposite the ovarian hilum. The ovary was gently removed from the bursa and the hilum clamped to prevent bleeding. The excised ovaries were placed in cold saline or digested to a single-cell suspension prior to transfer.

The FC/FD recipient mice underwent the same process to remove their endogenous, acyclic ovaries. After the endogenous ovaries were removed, young ovaries were placed into the recipient bursa through the original incision. The bursa was closed with sutures using 9-0 Ethicon monofilament (Ethicon, Inc.) and placed back into the body cavity. The abdominal wall was sutured with 6-0 Vicryl (Ethicon, Inc.), and the skin was closed using 9-mm wound clips (MikRon Precision, Inc.). The procedure was repeated on both ovaries in each recipient mouse. Mice were given analgesia and electrolytes and dextrose with continuous postoperative monitoring for any concerns until each mouse was bright, alert, and responsive. Postoperative mortality was less than 5%. OSC recipients underwent similar surgical procedures with the following exceptions: the endogenous postreproductive ovaries were left intact and injected with 10 ul of an OSC single cell suspension directly into both endogenous ovaries.

### Follicle (germ cell) depletion

We used 4-vinylcyclohexene diepoxide (VCD) to deplete germ cell-containing follicles in the ovaries of young mice [22]. Treatment with VCD selectively destroys primordial and primary follicles in the ovaries of rats and mice [36]. In contrast to ovariectomy, VCD treatment induces gradual ovarian failure through the depletion of the germ cell-containing follicles, while leaving the somatic ovary tissue intact. Germ cell-depleted mice are currently produced in our laboratory following the method of Lohff with minor modifications [37].

At 28 days of age, female mice received daily intraperitoneal (I.P.) injections of 160 mg/kg VCD in sesame oil for 20 days. FC ovary-donor mice were treated with sesame oil (vehicle) on the same schedule as the VCD-treated mice and stopped VCD and vehicle treatments at 48 days of age. At 60 days of age, ovaries were collected from VCD-treated and vehicle-treated mice for transplantation to 13-month-old, virgin CBA/J or 17-month-old virgin C57BL/6 mice. Ovaries from 60-day VCD-treated mice were also used for the isolation of young ovarian somatic cells for transplantation [22]. Primordial and primary follicle depletion is normally complete by day 46 after the initiation of VCD treatment, as reported previously in other strains of mice [38]. VCD-treated donor mice were superovulated prior to ovary collection to decrease any remaining secondary or later stage follicles. The mice were superovulated using 5 IU PMSG injected SC and subsequently received 5 IU hCG 46 h later.

### Cell isolation

Ovaries from FD 60-day mice were collected, digested, and somatic cells isolated as described previously [21]. Briefly, ovaries were surgically collected, moved to digestion media (DNase/Collagenase/Dispase) and minced with needles, incubated with frequent pipetting, centrifuged, and washed. Isolated somatic cells were cryopreserved for future use.

### Flow cytometry

To further dissect the mechanisms of the ovarian somatic cell-dependent restoration of health, two candidate ovarian somatic cell populations, ovarian surface epithelium (CK19+) and ovarian stroma (TEM-1+), were sorted from the total somatic cell isolate. Isolated somatic cells were tagged with cell-surface markers and sorted by flow cytometry as previously described [39]. Briefly, ovarian somatic cells were processed and immunostained using a TEM1/CD248 Polyclonal Antibody, a CK-19/TROMA-III-S monoclonal antibody and stained with a Fixable Live/Dead viability dye (Life Technologies). Flow cytometric analysis was performed using standard settings on a SORP FACSAria II Flow Cytometer (BD Biosciences). Samples were analyzed using FACSDiva Version 6.1.3. (BD Biosciences).

### Exosome collection

Exosomes from FD 60-day mice were collected from serum and isolated ovarian somatic cells. Cryopreserved sorted ovarian somatic cells were thawed and recovered in serum-containing media for 6 h, switched to serum-free media for 1 h then cultured for 24 h in fresh serum-free media. After 24 h, the media was collected. Exosomes were isolated from media and serum using the Vivaspin 500 Ultrafiltration Protocol for Extracellular Vesicle Isolation. Concentrated vesicles from media and serum were stored at −80 °C.

### Data-independent acquisition proteomics analysis

Data-independent acquisition (DIA) proteomics analysis was conducted in collaboration with the IDeA National Resource for Quantitative Proteomics. Briefly, frozen serum samples were sent to Dr. Samuel Mackintosh at the IDeA Center. Abundant serum proteins were depleted with HighSelect Top14 resin (Thermo) according to the manufacturer’s instructions. Proteins were reduced and alkylated prior to digestion with sequencing-grade modified porcine trypsin (Promega) using S-Trap columns (Protifi). Tryptic peptides were then separated by reverse phase XSelect CSH C18 2.5 um resin (Waters) on an in-line 150 × 0.075 mm column using an UltiMate 3000 RSLCnano system (Thermo). Peptides were eluted using a 60 min gradient from 98:2 to 65:35 buffer A:B ratio (Buffer A = 0.1% formic acid, 0.5% acetonitrile; Buffer B = 0.1% formic acid, 99.9% acetonitrile). Eluted peptides were ionized by electrospray (2.2 kV) followed by mass spectrometric analysis on an Orbitrap Exploris 480 mass spectrometer (Thermo). To assemble a chromatogram library, six gas-phase fractions were acquired on the Orbitrap Exploris with 4 m/z DIA spectra (4 m/z precursor isolation windows at 30,000 resolution, normalized AGC target 100%, maximum inject time 66 ms) using a staggered window pattern from narrow mass ranges using optimized window placements. Precursor spectra were acquired after each DIA duty cycle, spanning the m/z range of the gas-phase fraction (i.e., 496–602 m/z, 60,000 resolution, normalized AGC target 100%, maximum injection time 50 ms). For wide-window acquisitions, the Orbitrap Exploris was configured to acquire a precursor scan (385–1015 m/z, 60,000 resolution, normalized AGC target 100%, maximum injection time 50 ms) followed by 50 × 12 m/z DIA spectra (12 m/z precursor isolation windows at 15,000 resolution, normalized AGC target 100%, maximum injection time 33 ms) using a staggered window pattern with optimized window placements. Precursor spectra were acquired after each DIA duty cycle.

### Raman spectroscopy

Raman spectroscopy was performed as described previously [40]. Briefly, cells and exosomes were analyzed using a Renishaw inVia Raman spectrometer (Renishaw plc, UK) connected to a Leica microscope (Leica DMLM, Leica Microsystems, Buffalo Grove, IL, USA) that was used for the cell spectra collection. A 785-nm near-IR laser was equipped for the Raman spectrometer. The 50× dry lens objective in the spectral range 600 to 1800 cm^−1^ was implemented for the spectra collection. A silicon wafer was used for calibration before data collection (adjusted to 520.5 ± 0.1 cm^−1^ for the silicon peak). The exposure time was 10 s for 1 accumulation at 100% laser power density for all the cell samples. Cosmic rays in raw spectra were removed using “zap” function in Renishaw Wire 3.4 software. A volume of 20 µl of cells or EV sample was loaded on an MgF2 optical window and dried for 1 h at 37 °C before the spectra collection. For each EV type, spectra from three independent animals were collected (one optical window for each animal, total of 6 optical windows used). A total of 35 measurements were taken at each MgF2 optical window.

### Single-cell analysis

To identify putative TEM-1+ and CK19+ cell types, previously published single-cell RNA sequencing (scRNA-seq) data from adult murine ovarian cells (GSE232309; C57BL/6J mice, *n* = 8) were utilized (Fig. 6; Isola et al., 2024). The expression of Cd248 (TEM+) and Krt19 (CD19+) was visualized across previously identified distinct ovarian cell clusters using the FeaturePlot function from the Seurat R package, displayed over a UMAP (Uniform Manifold Approximation and Projection). Furthermore, the DotPlot function was employed to demonstrate the percentage of cells expressing these genes and their respective expression levels within each identified cell type.

### Gliosis

The severity of gliosis was assessed using immunohistochemical staining techniques previously described [41–43]. Briefly, gliosis was assessed in the brains collected from female WT controls and WT mice subjected to OSCs transplantation and from APP/PS1 mice. The severity of gliosis in tissue sections of the brain was measured using the immunohistochemical staining technique described by Bronson [42]. The severity of gliosis was graded as follows: Grade 0 (no changes), 1 (less than a 10% increase), 2 (10–25% increase), 3 (25–50% increase), and 4 (more than a 50% increase). These measurements of GFAP expression combined with pathological analysis allowed us to determine whether the progression of histopathological changes correlated with age- and treatment-related changes in other parameters.

### Open field

An open field test was conducted on young and old WT control mice and old WT-treated mice (FC, FD, and OSC). The FC, FD, and OSC groups produced similar results in all studies and were pooled (TX 22mo). As CBA/J strain female mice begin to experience irregular reproductive cycling relatively early, we included a 9-month group in the open field analysis to determine if minor cycling disruptions were linked to cognitive function and to model an age when women seeking to have children often experience difficulty conceiving [44–46]. We utilized an in-house 4-field, 3-quadrant apparatus monitored using a centrally placed camera and Any-maze® software. Tremor analysis was as described previously [22].

### Statistical analysis

Following data acquisition, protein data were searched using an empirically corrected library against the UniProt Mus musculus database (June 2021) and a quantitative analysis was performed to obtain a comprehensive proteomic profile. Proteins were identified and quantified using EncyclopeDIA [47] and visualized with Scaffold DIA using 1% false discovery thresholds at both the protein and peptide level. Protein MS2 exclusive intensity values were assessed for quality using ProteiNorm [48]. The data were normalized using cyclic loess [49] and analyzed using proteoDA to perform statistical analysis using Linear Models for Microarray Data (limma) with empirical Bayes (eBayes) smoothing to the standard errors [49, 50]. Proteins with an FDR adjusted *p*-value < 0.05 and a fold change > 2 were considered significant. For Raman spectroscopy, PCA was applied to examine the spectral differences between cells and cell and serum-derived EVs. The first ten PC scores were used in a supervised classification model, linear discriminant analysis (LDA), in order to discriminate and classify the data by maximizing the variance between groups. One-way analysis of variance (ANOVA) was applied for analyzing the difference of the characteristic spectral peaks among EVs. OriginPro 2018 (v. b9.5.1.195, OriginLab, Northampton, MA, USA) was used for data manipulation and analysis.

## Results

**Fig. 2:**
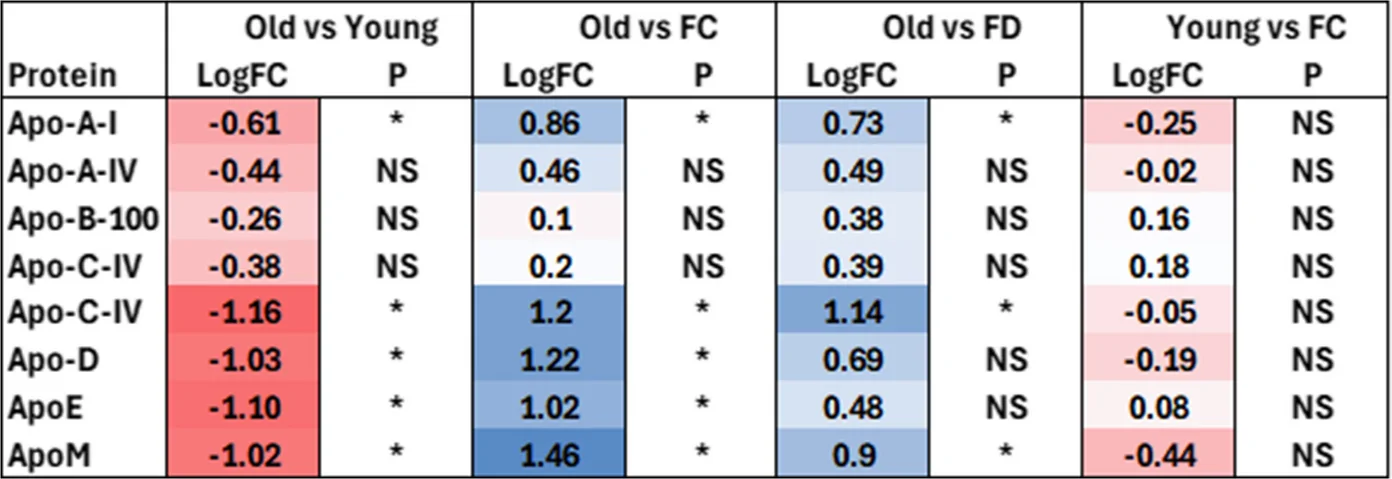
Change in apolipoprotein levels with age and ovarian tissue transplant. Apolipoprotein levels decreased with age from the levels in young 6mo mice to old, 17mo mice (DIA proteomics analysis is presented as LogFC in protein level compared with 6mo control mice). The age-associated decrease in apolipoprotein levels was reduced/reversed in old mice that received young ovarian tissue transplants, both with and without follicles (presented as LogFC in protein level compared with 17mo control mice). No significant difference was observed between young mice and old mice with young ovaries. Transplantations included: FC = intact, follicle-containing ovaries, and FD = follicle/germ-cell depleted ovaries. Excel

In the current study, our DIA proteomics analysis revealed changes in circulating apolipoprotein levels with age and with exposure to young ovarian tissues, both with transplantation of FC and FD ovaries. Apolipoproteins, including ApoA-I, ApoA-IV, ApoB-100, ApoC-I, ApoC-IV, AopD, AopE, and ApoM all decreased with age in postreproductive female mice (Fig. 2). Apolipoprotein levels in old mice that received follicle-containing young ovarian tissue transplants (FC) were increased from old controls and showed little change from the levels found in young mice. In old mice that received follicle-depleted young ovarian tissue transplants (FD), Apo protein levels again showed little change from levels in young mice (Fig. 2). While the direction of change in Apo protein levels was consistent between FC and FD treatments, some differences were noted between the magnitude of change in Apo levels in recipients that received FC young ovarian tissue and recipients that received FD young ovarian tissue.

**Fig. 3:**
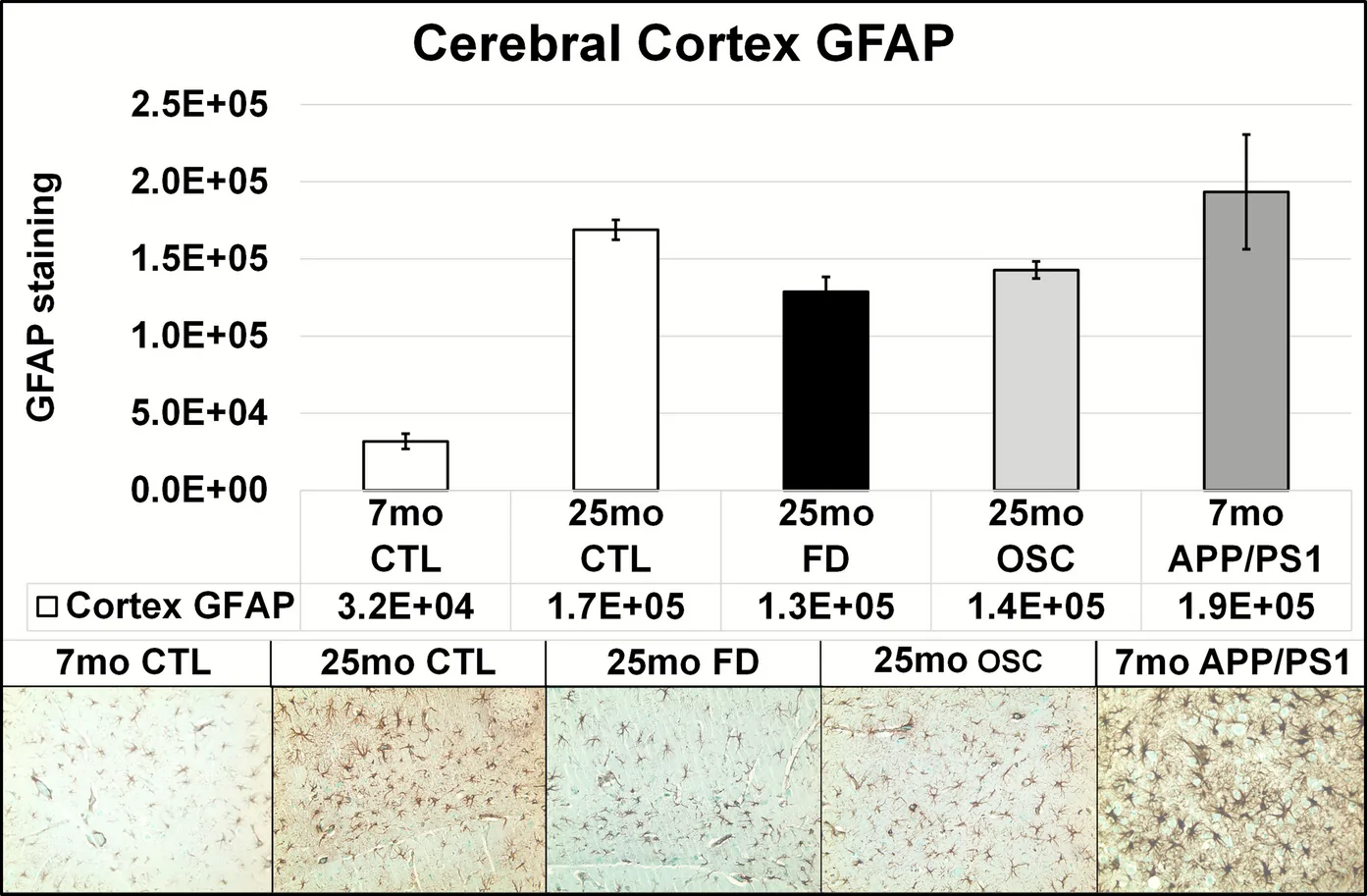
Analysis of GFAP staining in cerebral cortex tissue. Gliosis increased with age from 7 to 25mo in control mice. Gliosis was reduced in 25mo mice transplanted at the age of 18 months with whole ovaries after depletion of hormone-producing follicles (FD) or in 25mo mice transplanted with isolated ovarian somatic cells at the age of 18 months (OSC). Samples from 7-month APP/PS1 mice from a previous experiment are included for context (Dr. Ikeno). Excel

Transplantation of ovarian somatic tissue/cells also significantly influenced cognitive behavior and neuropathogenesis. Transplantation of ovarian somatic tissue/cells attenuated gliosis in aged female mice (Fig. 3). Gliosis was increased with age from 7 to 25 months in control WT mice and reduced in 25-month-old WT mice that received young ovarian tissue/cell transplants. Our current results are presented (in Fig. 3) alongside results from young APP/PS1 mice to provide context for the histological gliosis and quantitative GFAP staining in cerebral cortex tissues.

**Fig. 4:**
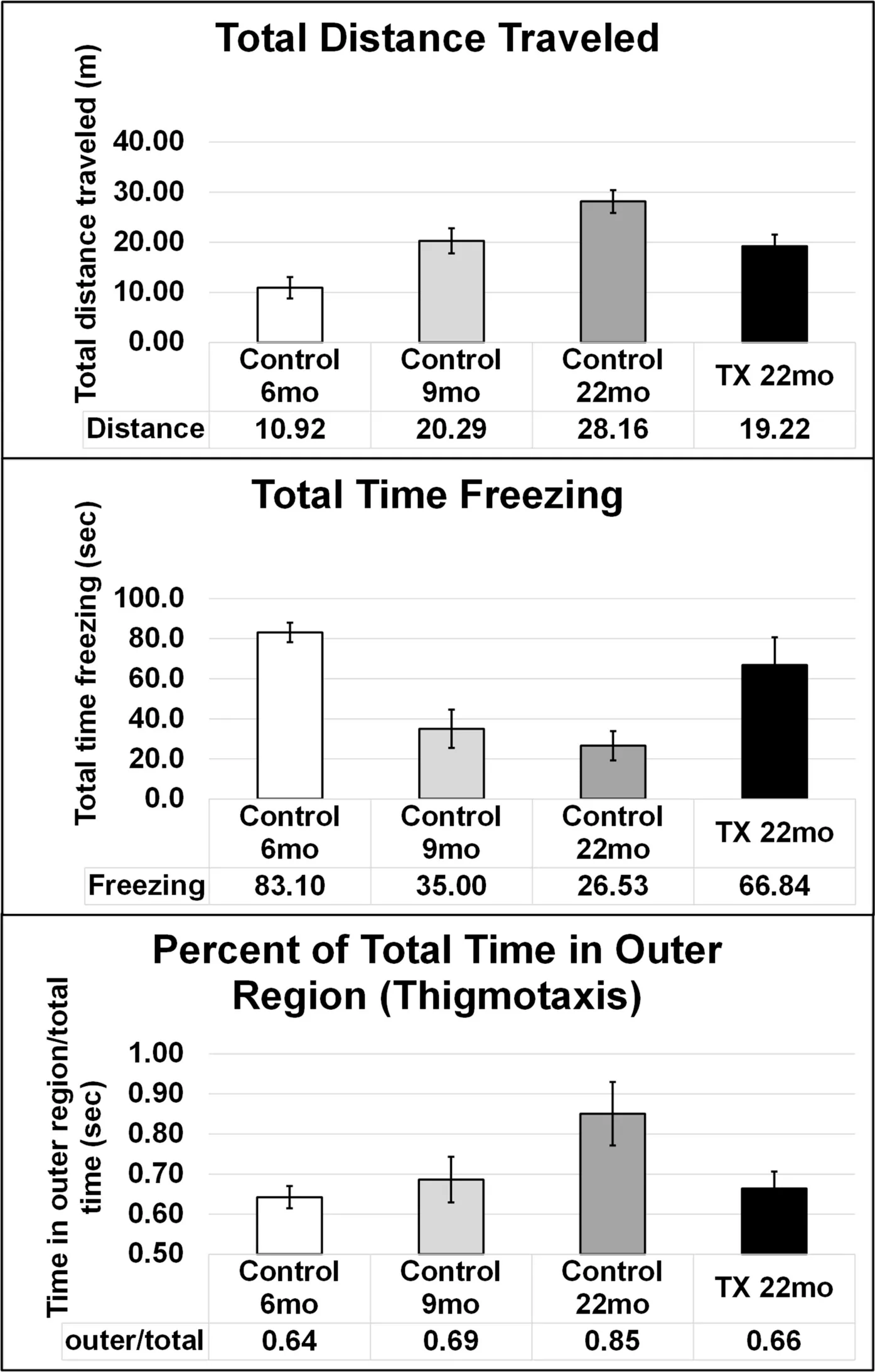
Open field analysis. Tremor amplitude was positively correlated with distance traveled in controls, but not in treated mice. Treated mice traveled the same distance, but had a decreased tremor amplitude (peak amplitude was 0.199 mV in transplant recipients vs. 0.308 mV in age-matched controls). Tremor amplitude was negatively correlated with total time freezing in both controls and treated mice. Treated mice had similar freezing time and tremor amplitude as young mice. Thigmotaxis, a measure of anxiety was decreased in transplant recipients (Tx = pooled FC, FD, and OSC transplanted mice). Excel

In the current open field analysis, total distance traveled increased with age in control mice, but not in transplanted mice. Open field freezing behavior also decreased with aging in our old wild-type mice, but was reversed in old transplant recipients to levels found in young WT mice. Transplantation of ovarian somatic tissue also significantly attenuated thigmotaxis/anxiety (Fig. 4).

**Fig. 5:**
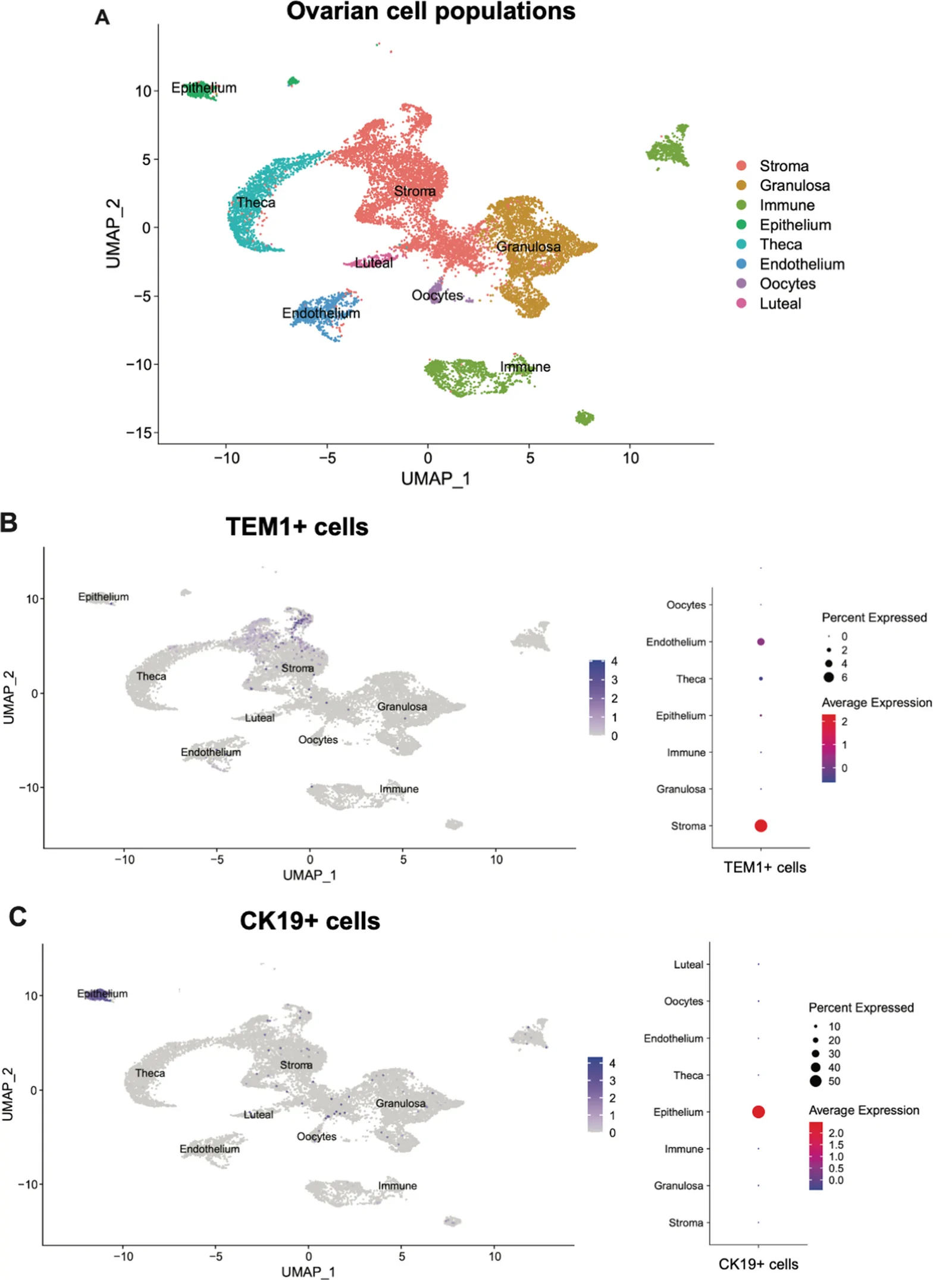
UMAP plot of murine ovarian cells demonstrating different cell types. **B** TEM1+ cells and **C** CK19+ cells were identified within the stromal and epithelial cell clusters, respectively. TEM1+ cells accounted for approximately 6% of the stromal cell cluster, while CK19+ cells comprised around 50% of the epithelial cell cluster. Excel

**Fig. 6:**
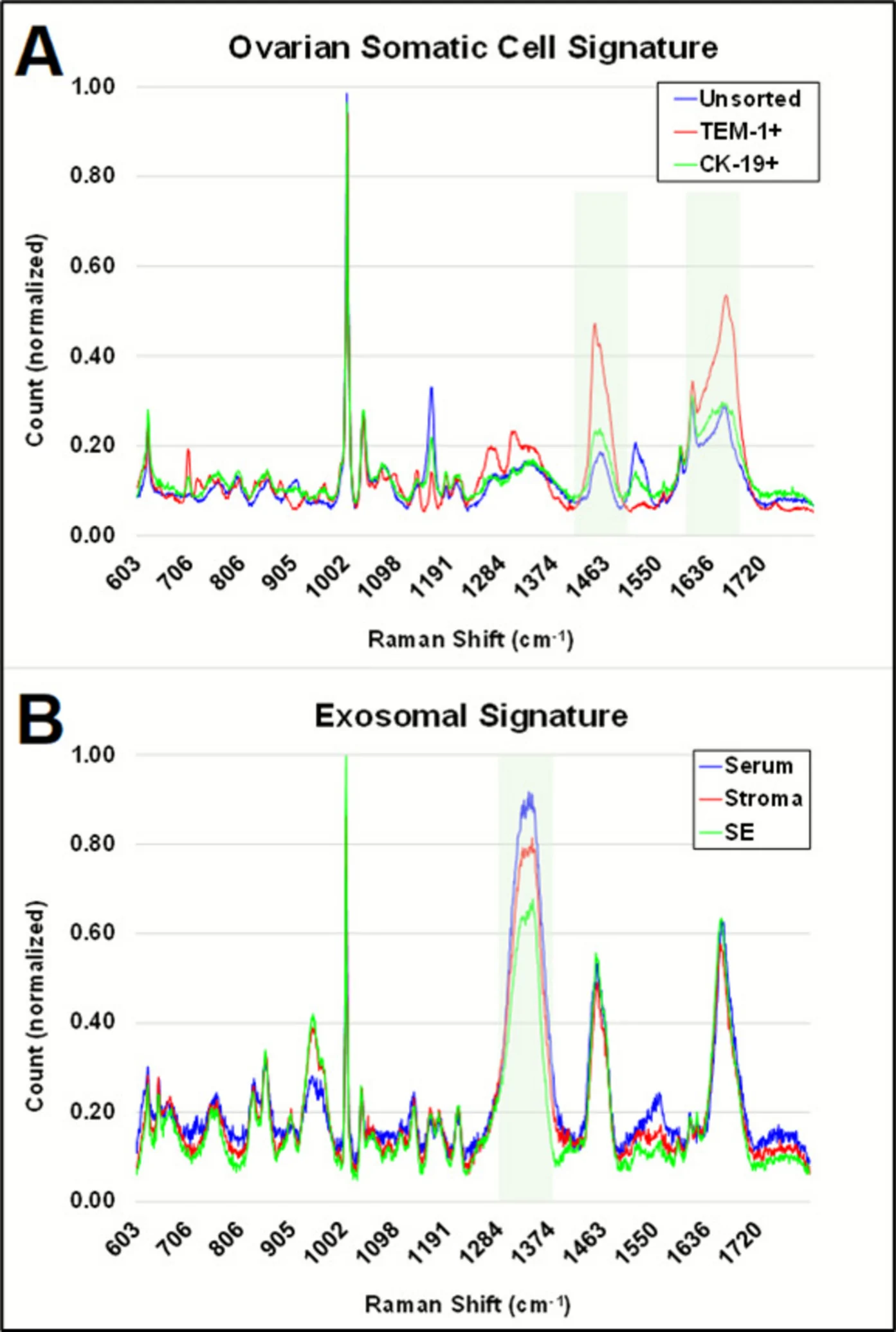
Pilot Raman spectroscopy of flow-sorted ovarian somatic cells and cell-type specific exosomes. **A** TEM-1+ cells (ovarian stroma) clearly separated from ovarian surface epithelium (CK-19+) and unstained cells on the basis of fatty acid saturation level. **B** Exosomes were also separated by ovarian somatic cell type and source (ovary vs. serum). SE, surface epithelium. Excel

Single-cell clustering analysis of isolated young ovarian somatic cells revealed that the two candidate ovarian somatic cell populations, ovarian surface epithelium (CK19+) and ovarian stroma (TEM-1+), were indeed distinct in their transcriptomic signatures and were segregated into epithelial (CK19) and stromal (TEM-1) clusters as predicted (Fig. 5). Pilot Raman spectroscopy of these ovarian somatic cells revealed that flow-sorted TEM-1+ cells clearly separated from other cell types based on lipid signatures. Raman spectra clearly indicate two strong, well-defined bands associated with lipid saturation levels across three primary cell groups at 1445 (CH2 bending of saturated fatty acids) and 1662 cm^−1^ (C=C bending of unsaturated fatty acids) (Fig. 6A). Exosomes isolated from the sorted cells and serum also displayed a similar separation based on lipid signatures suggesting the presence of a potential lipid-focused exosomal signaling mechanism (Fig. 6B).

## Discussion

Apolipoproteins are crucial for lipid metabolism and cognitive function, which includes memory, anxiety, attention, reasoning, and motor skills. ApoE influences cholesterol metabolism both in the brain and periphery [51]. In our study, we measured ApoE protein levels without identifying specific genetic isoforms. Previous research indicates that ApoE2 astrocytes and microglia secrete three to five times more ApoE protein than ApoE4 cells, with ApoE3 levels being intermediate [52]. This suggests that protein levels of ApoE significantly impact its function in the brain. For instance, ApoE2 mice show higher protein levels than ApoE4 mice [53], and neonatal ApoE3 mice have notably higher levels than ApoE4 counterparts [54]. Increased ApoE may help better regulate inflammatory signals in the brain, as demonstrated in various in vivo and in vitro models [54–59].

ApoE isoforms arise from cysteine-arginine exchanges; ApoE4 lacks cysteine, while ApoE2 has the most [60]. In Alzheimer’s disease (AD), oxidative stress creates neurotoxic molecules that bind to cysteine on ApoE2 and ApoE3. Without cysteine, ApoE4 cannot scavenge these toxins, possibly increasing oxidative damage [61]. Cysteine availability also affects glutathione synthesis [62]. Individuals with mild cognitive impairment (MCI) and AD show lower glutathione levels and GSH/GSSG ratios [63]. Restoring glutathione signaling may enhance cognitive outcomes.

We observed a significant decline of ApoA levels with age which was restored by ovarian transplant with and without follicles. Apolipoprotein A-I (ApoA-I) influences Alzheimer’s disease (AD) by binding to amyloid beta (Aβ). Serum ApoA-I levels are significantly lower in AD patients compared to healthy controls [64]. In our study, mice receiving ovarian tissue transplants exhibited restored circulating ApoA-I and ApoE and displayed improved cognitive function, reduced inflammatory signaling, and decreased gliosis in brain tissues ([19, 20, 22, 65]; Figs. 3 and 4).

We observed a significant decline of ApoD levels with age, which was restored by ovarian transplant with and without follicles. Loss of mouse ApoD function increases the sensitivity to oxidative stress and the levels of brain lipid peroxidation and impairs locomotor and learning abilities. Human ApoD overexpression in the mouse brain produces opposite effects, increasing survival and preventing the rise of brain lipid peroxides after oxidant treatment [66].

ApoC-1 levels appear to follow similar age (decreased) and transplant-associated (increased) changes, but not to the same degree as ApoC-4 and did not reach significant levels of change. ApoC-4 significantly declined with age and was restored by ovarian transplant. Apolipoprotein C1 (ApoC1) is associated with lipid metabolism and is a strongly associated risk factor for Alzheimer’s disease [67–70]. In humans, total ApoC1 levels are positively associated with plasma triglycerides and HDL [71], and ApoC1 can inhibit ApoE binding to the low-density lipoprotein receptor-related protein (LRP) [72, 73].

Combining our previous tremor work [22] with the current open field analysis revealed a correlation between total distance traveled and tremor amplitude in control mice, but not in transplanted mice, suggesting behavioral changes outweighed physical ability. Tremors are common in aging, with muscle quality declines being gender-specific and influenced by menopause. Our previous work demonstrated improved motor function and cognitive behavior in old mice post-ovarian transplant, indicating that young ovaries provide factors that may slow aging and prevent dementia [19, 22, 65]. These benefits appear independent of cyclic hormone production, supporting an additional, non-hormonal influence on the observed health benefits. Open field freezing behavior decreased with aging in wild-type mice but was restored in postreproductive female transplant recipients to levels seen in young mice. Additionally, transplantation significantly reduced thigmotaxis/anxiety. In a triple transgenic mouse model predisposed to ADRD, female mutants showed increased anxiety [74].

Gliosis is often used as a measure of neurodegeneration and is significantly increased in the APP/PS1 transgenic Alzheimer’s mouse model and is correlated with open field parameters [75]. Reactive gliosis can be a major contributor to cognitive impairment. In the current study, we demonstrated that gliosis can be detected in control (non-transgenic) aged animals and can be reduced by exposure to young ovarian tissues. Interestingly, while the direction of change in apolipoprotein levels was consistent between FC and FD treatments, differences noted between the magnitude of change in Apo levels in recipients that received FC young ovarian tissue and recipients that received FD young ovarian tissue or isolated ovarian somatic cells (OSCs) and changes in detection or prevention of gliosis between these two groups may suggest an indirect connection between the differentially regulated apolipoproteins and aging-associated gliosis.

Our single-cell analysis enabled the identification and characterization of different cell populations within our heterogeneous total ovarian somatic cell isolation through transcriptomics. Single-cell analysis revealed the predominance of TEM1+ cells being centered in the stromal cell clusters, while CK19+ accounted for nearly half of the epithelial clusters. Isolating these cell clusters can help target age-related lipid profiles in the various animal models. Accurate cell subpopulation identification was essential for verifying our flow cytometry and Raman spectroscopy targets.

Raman spectroscopy of the unsorted and flow-sorted ovarian somatic cells revealed that TEM-1+ cells (ovarian stroma) clearly separated from other cell types (surface epithelium CK-19), similar to our single-cell results, but now based on lipid signatures. Raman spectra clearly indicate two strong, well-defined bands associated with lipid saturation levels across cell groups at 1445 (CH2 bending of saturated fatty acids (Fas)) and 1662 cm^−1^ (C=C bending of unsaturated FAs). The 1660/1445 intensity ratio can be calculated to evaluate the degree of unsaturation of lipids [76].

The female-specific relationship between FA saturation and longevity suggests lower lipid peroxidation and fewer inflammatory precursors contribute to the extended longevity observed [77, 78]. Lipid profiling showed female-specific lipid changes associated with AD. Specifically, women with AD had fewer highly unsaturated lipids and more saturated lipids [79]. However, the relationship between FA saturation and longevity is tissue specific, sex linked, and species dependent [78].

We originally demonstrated that transplanting whole ovaries (follicle-containing; FC/GC) from 60-day-old mice extended health and longevity in postreproductive aged mice at 11, 13, 17, and 18 months of age. We further demonstrated, using follicle-depleted/germ cell-depleted ovary transplants (FD/GD transplants), that the restoration of health was not dependent on germ cell/follicle hormones, but on other putative pro-longevity factors that need future in-depth studies. Health parameters were further improved with intraovarian transplantation of isolated, young ovarian somatic cells (OSC) into the endogenous, postreproductive ovaries of aged females (i.e., T-cell function, and motor function, [22]). The origins of somatic cells in the ovary are complex, with multiple potential contributors to the somatic cell lineages [80]. Identifying the functional roles of these candidate somatic cell populations and connecting the molecular pathways with potential survival processes should ultimately determine the identities of health-enhancing ovarian cells. The disclosure of divergent transcriptomic signatures from our single-cell analysis and divergent lipid fingerprints from our Raman spectroscopy supports divergent roles for two cell types (CK19 and TEM-1) to be significant contributors to the demonstrated ovarian-dependent restoration of health in postreproductive females.

A limitation of the current study was our inability to demonstrate the long-term survival and integration of the transplanted ovarian somatic cells postoperatively. In a previous pilot experiment, ovarian somatic cells from a GFP+ female were injected into a GFP- ovary, and GFP+ cells were detected 1 week postoperatively. As a surrogate measure of the viability and longevity of the injected cells, we refer to the positive health span influence demonstrated by OSC-injected mice. In previous transplantation experiments, we recovered ovarian tissues from germ cell-containing and germ cell-depleted ovarian transplant recipients at the time of death, which were relatively indistinguishable from ovaries collected from control mice, indicating that the transplanted ovaries survived long term. While injecting isolated cells into an endogenous ovary is a very different procedure, the positive health span influence observed in OSC-injected mice mirrored that seen in the germ cell-containing and germ cell-depleted ovarian transplant recipients, suggesting a beneficial effect from the surviving injected cells.

## Conclusions

In the current study, mice that have previously demonstrated ovarian-dependent improvements in health, including regulation of several markers of aging, displayed significant changes in the patterns of circulating apolipoprotein levels and cognitive health, suggesting a connection between the levels of circulating apolipoproteins and the ovarian-dependent “global” improvements in health observed. Specific ovarian somatic cell populations exhibited divergent lipid-signaling fingerprints in fatty acid saturation pathways, which may be associated with extended longevity, particularly in females. Dysregulation of lipid metabolism is a common occurrence in several, if not most, aging models and is commonly reversed in many longevity interventions and should be considered for inclusion as “an additional” hallmark of aging.

## Data Availability

The data are not publicly available; however, they can be obtained from the corresponding author upon request.
